# Measurement of Changes in the Emotional State of Patients with Cardiac Diseases and Use of Cardiac Devices

**DOI:** 10.21470/1678-9741-2022-0460

**Published:** 2023-09-19

**Authors:** Julio Kevin Matos Flores, Lizeth Jackelin Cabrera Ipurre

**Affiliations:** 1 Professional School of Human Medicine, Faculty of Human Medicine, San Juan Bautista Private University, campus Chincha, Peru

Dear Editor,

Dessotte et al.^[1]^ recently presented, in the Brazilian Journal of Cardiovascular Surgery, an interesting parallel between the emotional changes that take place in patients with coronary conditions who use different cardiac devices for their treatment, and other variables in consideration.

Pérez et al.^[2]^ refer that the heart is the most affected organ in chronic Chagas disease, and this eventually induces dilated cardiomyopathy with systolic-diastolic dysfunction, arrhythmias, and sudden cardiac death. The diagnosis of this disease was evaluated in the Dessotte’s study.

However, neither the context nor the results justify the reason for including this illness. We consider it would have been better to take a general view of cardiac disorders, as evidenced in other similar investigations^[3,4,5]^. In this sense, we suggest that the title should have been “Comparison of anxiety and depression symptoms in individuals with heart disease according to sex and type of cardiac device”.

Further on, Dessotte et al. indicate that the Hospital Anxiety and Depression Scale (HADS) is used to evaluate anxiety and stress symptoms, justifying their choice. However, in one of the works cited by the authors to recommend the use of HADS^[6]^, this tool is not used, but rather two others: the Short Form 36 Health Survey (or SF-36) and the Assessment of QUAlity of Life and RELated Events (or AQUAREL). This is obviously a contradiction.

On the other hand, the Methodology does not mention the test used to evaluate the mean implantation time of implantable cardioverter defibrillators and pacemakers, although the results show that these variables were evaluated^[1]^, which casts doubt on the accuracy of these data. Likewise, providing the information in the form of averages is useful, but it would have been more interesting to know the ranges in which these measurements were taken in order to better understand the results and to allow them to be used as a reference for future studies.
